# Protein Phosphorylation and Mineral Binding Affect the Secondary Structure of the Leucine-Rich Amelogenin Peptide

**DOI:** 10.3389/fphys.2017.00450

**Published:** 2017-06-29

**Authors:** Hajime Yamazaki, Elia Beniash, Yasuo Yamakoshi, James P. Simmer, Henry C. Margolis

**Affiliations:** ^1^Center for Biomineralization, The Forsyth InstituteCambridge, MA, United States; ^2^Department of Developmental Biology, Harvard School of Dental MedicineBoston, MA, United States; ^3^Department of Oral Biology, Center for Craniofacial Regeneration, McGowan Institute for Regenerative Medicine, University of PittsburghPittsburgh, PA, United States; ^4^Department of Biochemistry and Molecular Biology, School of Dental Medicine, Tsurumi UniversityYokohama, Japan; ^5^Department of Biologic and Materials Sciences, University of Michigan School of DentistryAnn Arbor, MI, United States

**Keywords:** amelogenesis, amelogenin, leucine-rich amelogenin peptide, secondary structure, FTIR, tooth enamel

## Abstract

Previously, we have shown that serine-16 phosphorylation in native full-length porcine amelogenin (P173) and the Leucine-Rich Amelogenin Peptide (LRAP(+P)), an alternative amelogenin splice product, affects protein assembly and mineralization *in vitro*. Notably, P173 and LRAP(+P) stabilize amorphous calcium phosphate (ACP) and inhibit hydroxyapatite (HA) formation, while non-phosphorylated counterparts (rP172, LRAP(−P)) guide the growth of ordered bundles of HA crystals. Based on these findings, we hypothesize that the phosphorylation of full-length amelogenin and LRAP induces conformational changes that critically affect its capacity to interact with forming calcium phosphate mineral phases. To test this hypothesis, we have utilized Fourier transform infrared spectroscopy (FTIR) to determine the secondary structure of LRAP(−P) and LRAP(+P) in the absence/presence of calcium and selected mineral phases relevant to amelogenesis; i.e., hydroxyapatite (HA: an enamel crystal prototype) and (ACP: an enamel crystal precursor phase). Aqueous solutions of LRAP(−P) or LRAP(+P) were prepared with or without 7.5 mM of CaCl_2_ at pH 7.4. FTIR spectra of each solution were obtained using attenuated total reflectance, and amide-I peaks were analyzed to provide secondary structure information. Secondary structures of LRAP(+P) and LRAP(−P) were similarly assessed following incubation with suspensions of HA and pyrophosphate-stabilized ACP. Amide I spectra of LRAP(−P) and LRAP(+P) were found to be distinct from each other in all cases. Spectra analyses showed that LRAP(−P) is comprised mostly of random coil and β-sheet, while LRAP(+P) exhibits more β-sheet and α-helix with little random coil. With added Ca, the random coil content increased in LRAP(−P), while LRAP(+P) exhibited a decrease in α-helix components. Incubation of LRAP(−P) with HA or ACP resulted in comparable increases in β-sheet structure. Notably, however, LRAP(+P) secondary structure was more affected by ACP, primarily showing an increase in β-sheet structure, compared to that observed with added HA. These collective findings indicate that phosphorylation induces unique secondary structural changes that may enhance the functional capacity of native phosphorylated amelogenins like LRAP to stabilize an ACP precursor phase during early stages of enamel mineral formation.

## Introduction

Tooth enamel, the most highly mineralized tissue in the human body (>95 wt% mineral content), is comprised of intricate interwoven patterns of extremely long and narrow crystals of carbonated hydroxyapatite, which contribute to its exceptional functional capabilities. This extremely well-organized structure is established through highly-regulated extracellular processes during the secretory stage of amelogenesis (Nanci, 2013). During this stage where initial enamel mineralization takes place, amelogenin, the predominant protein component of the enamel matrix (>90%), is believed to play a major role in regulating the nucleation, growth, morphology, and organization of forming enamel crystals (Margolis et al., 2014). Full-length amelogenin (with 173 amino acids in porcine enamel) is comprised of a tyrosine-rich N-terminal domain that includes its only post-translational modification (phosphorylation) site at serine-16 (Ser-16), a large hydrophobic central domain, and a highly conserved hydrophilic C-terminal domain. Amelogenin has been shown to assemble into nano particles (nanospheres) or higher order chain-like structures under specific (including physiological) conditions (for reviews, see Fincham et al., 1999; Margolis et al., 2006). Previous studies also suggest this higher-order structure helps regulate calcium phosphate mineralization *in vitro* through cooperative interactions with forming mineral (Beniash et al., 2005), leading to the formation of crystalline arrays of mineral particles, similar to those found in developing enamel (Beniash et al., 2005; Kwak et al., 2009; Deshpande et al., 2010; Yang et al., 2010; Wiedemann-Bidlack et al., 2011). Importantly, recombinant non-phosphorylated amelogenins have been shown to transiently stabilize amorphous calcium phosphate (ACP) precursor phases *in vitro*, prior to their spontaneous transformation to crystalline hydroxyapatite (HA) (Kwak et al., 2009, 2011, 2014, 2016; Yang et al., 2010; Wiedemann-Bidlack et al., 2011). A similar transformation of ACP to crystalline mineral has also been observed in developing enamel (Diekwisch, 1998; Beniash et al., 2009). Most notably, the single-site phosphorylation of amelogenin (porcine) has been shown to have a marked effect on calcium phosphate mineralization *in vitro*; that is, both full-length and truncated phosphorylated amelogenins have an enhanced capacity to stabilize ACP and prevent HA formation (Kwak et al., 2009, 2011, 2014; Wiedemann-Bidlack et al., 2011) in a concentration-dependent fashion (Kwak et al., 2009, 2014; Fang et al., 2013).

The leucine rich amelogenin peptide (LRAP) is an alternative-splicing product of the amelogenin gene expressed throughout enamel development (Yuan et al., 1996). For example, the 56 amino acid porcine LRAP is comprised of the first 33 N-terminal amino acids (including the phosphorylation site) and the last 23 C-terminal amino-acids (including the hydrophilic domain) of the full-length porcine amelogenin. Numerous attempts have been made to elucidate the physiological function of LRAP in enamel formation. It has been proposed to have roles as a cell signaling molecule (Veis et al., 2000; Boabaid et al., 2004; Warotayanont et al., 2008, 2009; Wen et al., 2011) or to be involved in the regulation of the kinetics of calcium phosphate mineralization and the morphology of formed crystals (Le Norcy et al., 2011a; Xia et al., 2016). However, a consensus regarding the roles of LRAP in amelogenesis has not been reached and still many questions remain unanswered. Nevertheless, previous studies have shown that LRAP shares many common properties with the full-length amelogenin with respect to its capacity to regulate mineral formation *in vitro*. Like full-length amelogenin, LRAP forms nanospheres (Habelitz et al., 2006; Tarasevich et al., 2010; Le Norcy et al., 2011a), and appears to interact with hydroxyapatite (Shaw et al., 2004, 2008). Furthermore, it has been shown that non-phosphorylated recombinant human LRAP and recombinant full-length human amelogenin (rH174) have the same capacity to bind calcium (i.e., four to six calcium ions per molecule), although the calcium affinity constant for the LRAP was greater than that for the full-length amelogenin (Le et al., 2006). We have also demonstrated that non-phosphorylated porcine LRAP (LRAP(−P)) can similarly guide the formation of aligned bundles of HA crystals, as does the recombinant non-phosphorylated amelogenin (Le Norcy et al., 2011a), while, like native phosphorylated versions of amelogenin, phosphorylated porcine LRAP (LRAP(+P)) similarly stabilizes ACP and prevents HA formation *in vitro*. Based on similarities of amino acid sequences and behaviors, LRAP has also allowed us to investigate the potential role of specific amino-acid domains of amelogenin and phosphorylation in protein self-assembly. Our previous study using dynamic light scattering (DLS) and transmission electron microscopy (TEM) illustrates that there are potentially important differences in the self-assembly and conformational behavior between phosphorylated LRAP(+P) and its non-phosphorylated counterpart, LRAP(−P) (Le Norcy et al., 2011a). Also, previous studies from our laboratory using small angle X-ray scattering (SAXS) techniques showed dramatic structural differences between LRAP(+P) and LRAP(−P) that are further affected by the presence of calcium ions (Le Norcy et al., 2011b). We are specifically interested in the role the single phosphate group in amelogenin plays in enamel mineral formation and have hypothesized that phosphorylation of amelogenin induces conformational changes that critically affect its capacity to interact with forming calcium phosphate mineral phases. To test this hypothesis, we have utilized Fourier transform infrared spectroscopy (FTIR) in the present study to ascertain the effect of phosphorylation on the secondary structures of LRAP(−P) and LRAP(+P) in the presence and absence of calcium in solution and upon interacting with relevant mineral phases (i.e., HA and ACP). FTIR spectroscopy is extremely sensitive to global conformational changes in proteins (Surewicz et al., 1993; Wang et al., 2010) and uniquely suited to study structural changes in proteins upon self-assembly (Bouchard et al., 2000; Wang et al., 2010) and adsorption to solid surfaces (Roach et al., 2005; Elangovan et al., 2007).

## Materials and methods

### Preparation of LRAP solutions

Porcine LRAP with or without a phosphate group on Ser-16 [i.e., LRAP(+P) and LRAP(−P), respectively] were synthesized commercially (RS Synthesis, Louisville, KY, USA) and re-purified by high-pressure liquid chromatography (HPLC), as previously described (Nagano et al., 2009). Lyophilized LRAP(+P) and LRAP(−P) were weighed and dissolved in distilled deionized water at room temperature to yield stock solutions of 17.5 mg/mL (pH 2.5 ~ 3) solutions. The stock solutions were left for 12–24 h at 4°C to aid complete protein dissolution. Stock solutions were then stored at −20°C. Just prior to use, aliquots of the LRAP stock solutions were centrifuged (12,500 × g, 4°C, 20 min) and the supernatants were diluted to 15 mg/mL with either distilled deionized water or calcium chloride solution to yield 7.5 mM calcium. The pH of each solution was adjusted to pH 7.4 at room temperature using potassium hydroxide aqueous solution. Each experimental solution type [i.e., LRAP(−P) and LRAP(+P), with and without added calcium] was prepared in the same fashion in triplicate (*n* = 3).

### Dynamic light-scattering (DLS) measurements of LRAP solutions

To acquire information on the aggregation of LRAP, each solution type was subjected to dynamic light-scattering (DLS) analysis, as previously described (Wiedemann-Bidlack et al., 2007). Each DLS measurement (DynaPro MSXTC/12) was comprised of 5 measurements of 20 acquisitions (5 sec each) at 5-min intervals at 25°C and the sizes (hydrodynamic radius, R_H_) of protein particles were determined. Unpaired *t*-tests were used to compare differences in protein particle sizes.

### Incubation of LRAP(+P) and LRAP(−P) with selected mineral phases

Standard HA was purchased from National Institute of Standards and Technology (2910 Calcium Hydroxyapatite, Gaithersburg, MD, USA). Stabilized ACP was prepared by mixing CaCl_2_ and NaH_2_PO_4_ in distilled water to final concentrations of 5 and 3 mM, respectively, at ambient conditions with stirring in the presence of 150 μM of Na_4_P_2_O_7_. After 60 min, the reaction suspension was centrifuged at 12,000 × g at 4°C for 20 min. The pellets were washed with distilled water twice, lyophilized, and stored at −20°C. The composition and structure of the standards were confirmed using FTIR prior to use. The stability of the ACP phase in water was also confirmed by FTIR after incubation in water for 4 h at 37°C, following the experimental protocol described in the next paragraph. These latter selected measurements were carried out at Emmanuel College in Boston, MA (see Acknowledgments).

HA or ACP (0.3 mg) were incubated in 40 μL of 5, 10, and15 mg/mL LRAP(+P) and LRAP(−P) solutions with rocking for 4 h at 37°C. After equilibration, the mineral-protein mixtures were centrifuged at 12,000 × g for 10 min at 4°C. After centrifugation, the supernatants were removed, and the pellets were washed twice (10 min each) with 20 μL of distilled deionized water (pH adjusted to 7.4). The washed samples with bound protein were then re-suspended in 10 μL of the pH-adjusted distilled deionized water, and the suspensions were used for FTIR measurements. In this fashion, the effect of the binding of LRAP(+P) and LRAP(−P) to HA and ACP on protein secondary structure were assessed, as was done similarly for full-length amelogenin (Beniash et al., 2012).

### FTIR spectroscopic measurement of LRAPs in the absence and presence of calcium and following equilibration with mineral particles

FTIR spectroscopic measurements were conducted at room temperature, as previously described (Elangovan et al., 2007; Beniash et al., 2012), using the attenuated total reflection (ATR) mode. Fifteen microliters of protein solution or washed mineral suspension were placed within a small rubber O-ring (i.d., 3 mm) on the ATR crystal. The sample was then covered with a glass slide that was pressed down with the ATR accessory press against the O-ring to minimize evaporation. Sample and background (distilled deionized water) spectra were taken at a resolution of 4 cm^−1^, and 128 scans were collected *per* spectrum.

### FTIR spectra analyses

Analyses were performed using the Origin 9.0 software package (OriginLab Corporation, Northampton, MA), as previously described (Elangovan et al., 2007; Beniash et al., 2012). For LRAP(−P) and LRAP(+P) in the presence and absence of calcium ions, FTIR spectra were measured three times for each solution, and the averaged spectra of the triplicate measurements were used for the further analyses. For suspensions of LRAP(−P) or LRAP(+P) with the mineral particles (HA or ACP), however, only data from the experiment with 15 mg/mL LRAP were used, since the spectra obtained with the 5 and 10 mg/mL solutions were too noisy for reliable deconvolution analyses (described in the following paragraph). However, the observed tendency in differences of spectra from LRAP(+P) and LRAP(−P) at lower concentrations were the same as those seen in the experiments carried out with the highest concentration of each LRAP.

The amide I and amide II region (between 1,475 and 1,725 cm^−1^) of the spectra were smoothed (5-point FFT smoothing), baseline corrected (straight line subtraction from the start to end points). Second derivative analyses were then performed to obtain peak minima that were used to identify the initial center of the identified individual peaks. Peak-fitting was performed using a Gaussian model. Identified peak positions were initially fixed, and several rounds of peak-fitting were performed until χ^2^ values between the experimental and calculated spectra were reduced to a value below 1 × 10^−6^. The same procedure was then repeated with the peak center released with the restriction of movement of ± 2 cm^−1^ until χ^2^ values between the experimental and calculated spectra were reduced to a value below 1 × 10^−6^. The percentage of the each deconvoluted peak area within the peak area of the amide I region (between 1,600 and 1,700 cm^−1^) was then calculated for each spectrum. Identified peaks within the amide I region were then attributed to specific secondary structural elements, as described in the next paragraph.

### FTIR peak assignments

FTIR peak identifications were based on the following literature reports. Peaks observed at 1,620–1,630 cm^−1^ were identified as hydrated PPII helix (Johnston and Krimm, 1971; Wellner et al., 1996; Elangovan et al., 2007). Earlier reports indicate that the full length amelogenins contain a significant PPII fraction (Renugopalakrishnan et al., 1986; Goto et al., 1993; Sogah et al., 1994; Lakshminarayanan et al., 2007, 2009). In an overlapped region to this, peaks observed between 1,610 and 1,640 cm^−1^ were attributed to β-sheet (Susi and Byler, 1983; Jackson and Mantsch, 1995). Random coil conformation was attributed to peaks between 1,640 and 1,650 cm^−1^ (Krimm and Bandekar, 1986; Barth and Zscherp, 2002; Elangovan et al., 2007), which have also been reported in amelogenin (Renugopalakrishnan et al., 1986; Goto et al., 1993; Matsushima et al., 1998; Elangovan et al., 2007; Yang et al., 2010). Also, peaks observed between 1,650 and 1,655 cm^−1^ were attributed to α-helix conformation (Susi and Byler, 1983; Surewicz et al., 1993; Roach et al., 2005). Finally, peaks observed between 1,659 and 1,670 cm^−1^ were assigned to β-turn (Susi and Byler, 1983; Surewicz et al., 1993; Jackson and Mantsch, 1995; Vass et al., 2003). A later peak with a maximum around 1,680 and 1,690 cm^−1^ can also be attributed to β-turn or high-frequency split of the anti-parallel β-sheet (Krimm and Bandekar, 1986; Kubelka and Keiderling, 2001; Elangovan et al., 2007).

## Results

### DLS measurements of LRAP in solution

Mean protein particle sizes from DLS measurements (S.D.) in the absence [LRAP(−P): 5.55 (0.18) nm; LRAP(+P): 3.87 (0.61) nm] and presence [LRAP(−P): 5.07 (0.57) nm; LRAP(+P): 5.51 (0.59) nm] of 7.5 mM calcium at pH 7.4 confirmed that both non-phosphorylated and phosphorylated LRAP undergo self-assembly to form small nanoparticles under near-neutral pH conditions, as we have previously reported (Le Norcy et al., 2011a). LRAP(+P) exhibits a smaller particle size (*p* < 0.0005) in comparison to LRAP(−P). In addition, the LRAP(+P) particle size increases significantly (*p* < 0.00005) in the presence of added calcium, while the particle size of LRAP(−P) changed only slightly (*p* < 0.05). These latter results on the effect of calcium on LRAP particle size are consistent with our earlier findings (Le Norcy et al., 2011a).

### FTIR analyses of the secondary structure of LRAP(−P) and LRAP(+P) in the presence/absence of calcium ions

**Figure 1:**
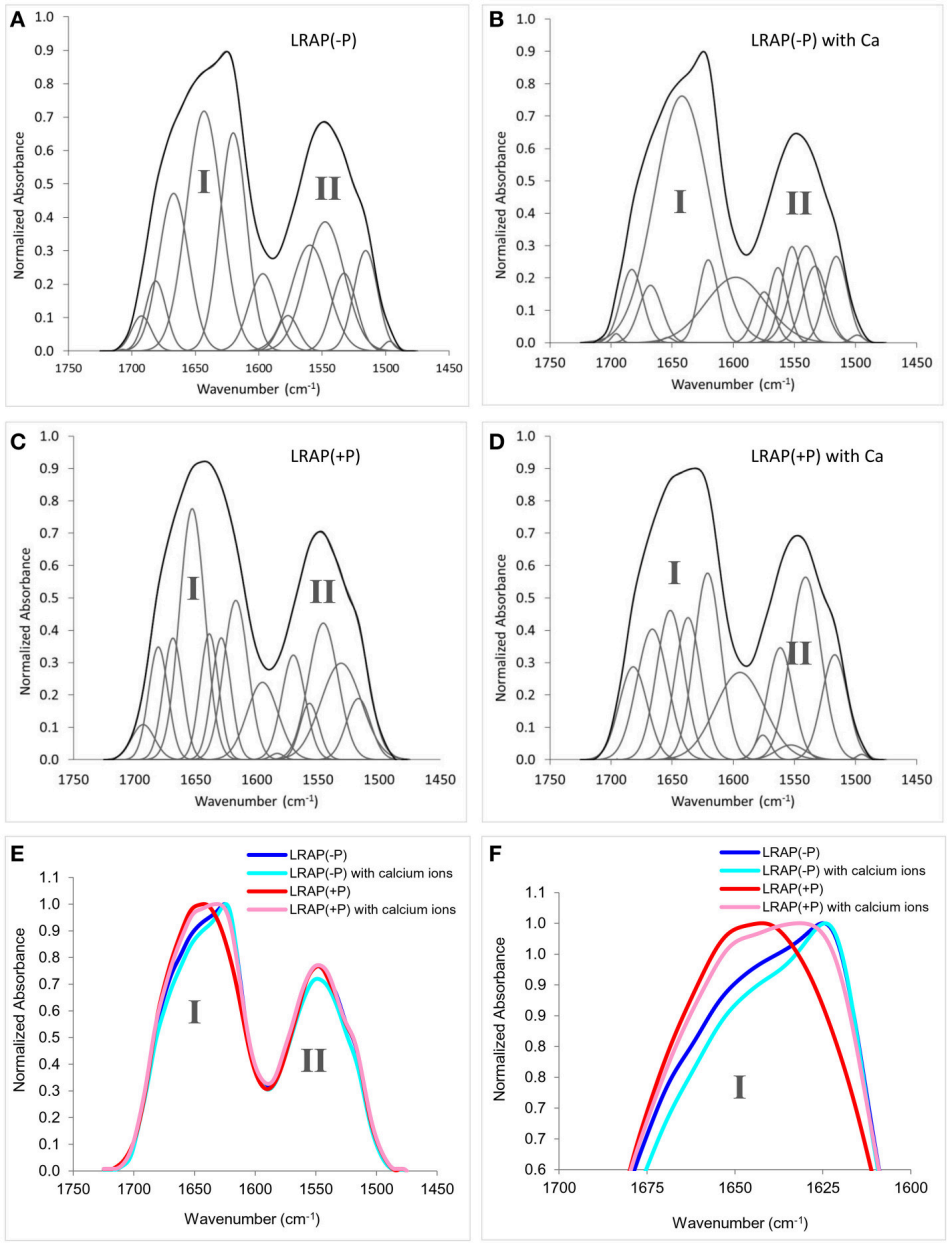
Amide I and amide II regions of FTIR spectra and individual fittings, showing deconvoluted peaks of LRAPs in the presence or absence of calcium ions., Amide I (1,700–1,600 cm−1) and amide II regions (1,575–1,480 cm−1) are labeled “I” and “II”, respectively. **(A)** LRAP(−P), **(B)** LRAP(−P) in the presence of calcium ions, **(C)** LRAP(+P), **(D)** LRAP(+P) in the presence of calcium ions. **(E)** Superimposed plotting of all 4 aforementioned spectra in **(A–D)**. **(F)** Expanded view of the upper portions of amide I peaks shown in **(E)**.

Figures 1A–D show amide I and amide II regions of the FTIR spectra (1,475–1,725 cm^−1^) and individual deconvoluted peaks obtained after peak analyses for LRAP(−P), LRAP(−P) with calcium ions, LRAP(+P), and LRAP(+P) with calcium ions, respectively. Figures 1E,F show the 4 mean spectra superimposed in the same plot for comparative purposes. Also, the results of the peak analysis are summarized in Table 1, as a list of peak positions (represented as wavenumbers of the individual peak centers) and the area percentage of the individual peaks identified within the amide I region, obtained from each deconvoluted peak. As shown in Figure 1A and Table 1, non-phosphorylated LRAP(−P) is mostly comprised of a 1,643 cm^−1^ peak (40%) that is attributed to random coil and a peak at 1,620 cm^−1^ (28%) that is attributed to PPII helix or β-sheet structure. In the presence of calcium ions with LRAP(−P), this β-sheet/PPII helix component at 1,620 cm^−1^ is significantly reduced (to 8.8%), and the overlapping major peak associated with random coil structure at 1,642 cm^−1^ increases in total area (*to* 74.3%), as shown in Figure 1B and Table 1. However, the overall change of the LRAP(−P) spectra upon addition of calcium is relatively subtle and the overall shape of the amide I peak of LRAP(−P) remains fairly similar (see Figure 1F) with the highest absorbance remaining at ~1,620 cm^−1^. As shown in Figure 1C and Table 1, however, phosphorylated LRAP(+P) exhibited evidence for three different β-sheet structures (total 42%) as multiple peaks (1,617, 1,629, and 1,639 cm^−1^), although the possibility of PPII helix components cannot be ruled out (i.e., 1629 cm^−1^). In contrast to the non-phosphorylated LRAP(−P) in the absence or presence of calcium, however, LRAP(+P) lacked random coil structure and exhibited a significant amount of α-helix (1,653 cm^−1^, 31.4%) as a major secondary structure component. In the presence of calcium ions, the amide I peak of LRAP(+P) exhibited a decrease in α-helix conformation (from 31.4% to 21.2% at 1,652 cm^−1^), along with notable increases in β-turn (1,666 cm^−1^), β-sheet (1,637 cm^−1^), and formed β-sheet/PPII helix components shown (1,620 cm^−1^) (see Figure 1D and Table 1). As shown in Figures 1E,F, with the addition of calcium ions, the overall shape of the LRAP(+P) amide I peak changes dramatically, shifting its peak absorption frequency from 1,650 cm^−1^ toward 1,620 cm^−1^, consistent with more significant changes in LRAP(+P) secondary structure, in comparison to that seen with the non-phosphorylated LRAP(−P).

**Table 1:** Positions and relative areas of individual deconvoluted peaks within amide I region of FTIR spectra of LRAP(−P) and LRAP(+P) in the presence or absence of calcium ions.

	**LRAP(–P)**		**LRAP(–P) with Ca**		**LRAP(+P)**		**LRAP(+P) with Ca**	
	**Center (cm^−1^)**	**Area%**	**Center (cm^−1^)**	**Area%**	**Center (cm^−1^)**	**Area%**	**Center (cm^−1^)**	**Area%**
Anti-parallel β-sheet					1,617	19.3		
β-sheet or polyproline II helix	1,620	27.8	1,620	8.8			1,621	25.6
β-sheet or polyproline II helix					1,629	11.5		
β-sheet								
β-sheet					1,639	11.5	1,637	18.8
Random coil	1,643	39.7	1,642	74.3				
α-helix	1,652	0.0	1,654	0.2	1,653	31.4	1,652	21.2
β-turn or 3(10) helix								
β-turn or 3(10) helix	1,667	22.2	1,668	7.4	1,669	11.5	1,666	21.0
β-turn or β-sheet								
β-turn or β-sheet	1,681	6.9	1,683	9.0	1,680	10.8	1,682	13.4
β-turn or β-sheet	1,692	3.4	1,696	0.4	1,693	4.1	1,695	0.0

### FTIR spectroscopic analyses of the secondary structure of LRAP(−P) and LRAP(+P) in the presence of HA or ACP

**Figure 2:**
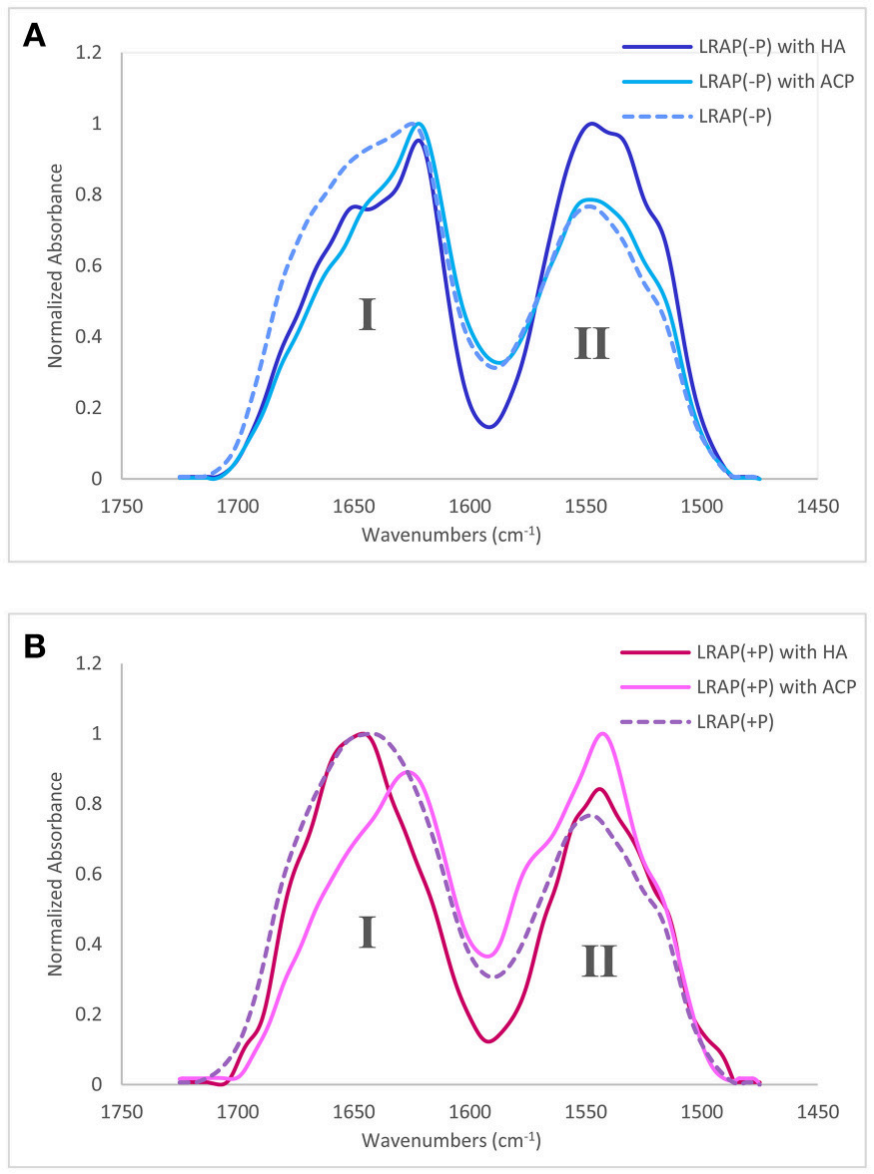
Amide I and amide II regions of FTIR spectra and individual fittings, showing deconvoluted peaks of LRAPs in the presence or absence of mineral particles. Amide I (1,700–1,600 cm−1) and amide II regions (1,575–1,480 cm−1) are labeled “I” and “II”, respectively. **(A)** Superimposed plots from three different experiments, LRAP(−P) (dotted line), LRAP(−P) in the presence of HA (dark blue), and LRAP(−P) in the presence of ACP (light blue). **(B)** Superimposed plot of three different experiments, LRAP(+P) (dotted line), LRAP(+P) in the presence of HA (dark red), and (LRAP(+P) in the presence of ACP (light pink).

Amide I and amide II areas of FTIR spectra (1,475–1,725 cm^−1^) of LRAPs, without mineral particles, with HA, and with ACP are shown in Figure 2A (LRAP(−P)) and Figure 2B (LRAP(+P)). Corresponding results of peak identification and analyses within the amide I region are summarized in Table 2, in the same manner as described above. As shown in Figure 2A, in comparison to the amide I peak of LRAP(−P) without minerals (dotted line), both the addition of HA and ACP mineral particles induced a significant relative increase in β-sheet/PPII helix structure at around 1,620 cm^−1^, along with a notable decrease in random coil structure at 1,643 cm^−1^ in comparison to that seen at higher wavenumbers. The amide I peak of LRAP(−P) incubated with HA also showed the formation of an α-helix component (1,650 cm^−1^), whereas amide I peak of LRAP(−P) incubated with ACP did not. In contrast to LRAP(−P), as shown in Figure 2B, observed changes in the secondary structure of LRAP(+P) showed a completely different pattern that also depended on the mineral phase in question, as can be clearly seen in Figure 2B. As summarized in Table 2, the amide I peak of LRAP(+P) incubated with HA showed a decrease in the α-helix component and an increase in random coil structure (1,641 cm^−1^), resulting in an amide I peak with a maximum absorption around 1,650 cm^−1^. On the other hand, the amide I peak of LRAP(+P) incubated with ACP showed a marked increase in β-sheet/PPII helix structure component at 1,619 cm^−1^, along with a slight decrease (31–26%) in the α-helix component at 1,653 cm^−1^. These collective changes resulted in the maximum absorption in the amide I band shifting to ~1,620 cm^−1^ as was shown in the case of LRAP(−P) incubation with either mineral phase.

**Table 2:** Positions and relative areas of individual deconvoluted peaks within amide I region of FTIR spectra of LRAP(−P) and LRAP(+P) in the presence or absence of mineral particles (HA or ACP).

	**LRAP(–P)**		**LRAP(–P) with HA**		**LRAP(–P) with ACP**		**LRAP(+P)**		**LRAP(+P) with HA**		**LRAP(+P) with ACP**	
	**Center (cm^−1^)**	**Area%**	**Center (cm^−1^)**	**Area%**	**Center (cm^−1^)**	**Area%**	**Center (cm^−1^)**	**Area%**	**Center (cm^−1^)**	**Area%**	**Center (cm^−1^)**	**Area%**
Side chain									1,601	3.2		
Anti-parallel β-sheet							1,617	19.3	1,617	13.3		
β-sheet or polyproline II helix	1,620	27.8	1,622	42.6	1,621	53.7					1,619	47.8
β-sheet or polyproline II helix							1,629	11.5	1,628	7.3	1,632	13.6
β-sheet												
β-sheet			1,638	9.4			1,639	11.5				
Random coil	1,643	39.7			1,644	14.7			1,641	23.3		
α-helix	1,652	0.0	1,650	18.7			1,653	31.4			1,653	25.8
β-turn or 3(10) helix					1,661	21.7			1,659	28.5		
β-turn or 3(10) helix	1,667	22.2	1,664	15.4			1,669	11.5	1,669	10.3	1,667	5.5
β-turn or β-sheet												
β-turn or β-sheet	1,681	6.9	1,680	13.9	1,682	10.0	1,680	10.8	1,678	13.1	1,680	7.3
β-turn or β-sheet	1,692	3.4	1,694	0.0	1,695	0.0	1,693	4.1	1,695	1.1	1,695	0.0

## Discussion

Prior studies to investigate the secondary structure of amelogenin (summarized in Table 3) have led to a general consensus that amelogenin is an intrinsically disordered molecule, having a secondary structure that is mostly composed of random coil. Some reports also suggest that the N-terminus of amelogenin contains β-sheets, β-strand, β-turns, and α-helix components and that poly-proline type II (PPII) helical structure is found in the mid-region of amelogenin, while random coil conformation comprise the main part of the C-terminal domain. The secondary structure of LRAP has also been extensively studied (see Table 4 for summary and additional discussion). Some of these findings are discussed below. However, the general consensus is that LRAP is also an unstructured protein like full-length amelogenin, being comprised mostly of random coil, β-turn, and small amounts of helix structures, although LRAP is somewhat less structured in comparison to that proposed for the N- and C-terminal domains of full-length amelogenin (Delak et al., 2009; Zhang et al., 2011).

**Table 3:** Brief summary of previous reports on the secondary structure of amelogenin^*^.

**Authors**	**Type of amelogenin**	**Ser16 phosphorylation**	**species**	**Experimental techniques**	**Experimental conditions**	**Information obtained**
Renugopalakrishnan et al., 1986	Full-length	Native	Bovine	CD, FTIR (liquid and solid)	Various buffers at different pH values	Secondary structure
Goto et al., 1993	Full-length and cleaved (25, 20, and 13 kD)	Native	Porcine	CD	pH ~5, ~5 mM acetate	Secondary structure
Matsushima et al., 1998	Full-length and cleaved (20, and 13 kD)	Native	Porcine	SAXS, Computer modeling	2% acetic acid buffer	Secondary and tertiary structures
Lakshminarayanan et al., 2007	Full-length (rP172) and cleaved (rP148)	(−P)	Porcine (recombinant)	FTIR, CD, ITC, DLS	Tris buffer at pH 5.8	Secondary and quaternary structures, role of C-terminus
Delak et al., 2009	Full-length (rP172)	(−P)	Porcine (recombinant)	DLS, CD, NMR, computer simulation		Secondary structure
Lakshminarayanan et al., 2009	Full-length (rP172)	(−P)	Porcine (recombinant)	ITC, CD	Tris buffer, 5 mM Ca, pH 7.4, different temperatures	Secondary and quaternary structures
Lakshminarayanan et al., 2010	Full-length (rM180 with His-tag^*^, point mutation)	(−P)	Murine (recombinant)	DLS, CD, fluorescence spectrometry	acetate buffer, pH 5.8,	Secondary and quaternary structures—effects of selected point mutations
Zhang et al., 2011	Full-length (fragmented) with His-tag^*^		Murine (recombinant)	SS-NMR (REDOR), SV	Phosphate and acetate buffers	Secondary and quaternary structures
Beniash et al., 2012	Full-length (rP172)	(−P)	Porcine (recombinant)	FTIR	PBS, Ca	Secondary structure—effects of pH and added calcium

* *CD, Circular Dichroism; DLS, Dynamic Light Scattering; FTIR, Fourier Transform Infrared Spectroscopy; His-tag, 12 amino acid peptide tag (MRGSHHHHHHGS-); ITC, Isothermal Titration Calorimetry; NMR, Nuclear Magnetic Resonance; REDOR, Rotational Echo DOuble Resonance; SAXS, Small Angle X-ray Scattering; SS-NMR, Solid State Nuclear Magnetic resonance; SV, Sedimentation Velocity*.

**Table 4:** Brief summary of previous reports on the secondary structure of LRAP^*^.

**Authors**	**Type of amelogenin**	**Ser16 Phosphorylation**	**species**	**Experimental techniques**	**Experimental conditions**	**Information obtained**
Shaw et al., 2004	LRAP	(+P) and (−P)	Recombinant	SS-NMR (REDOR)	Phosphate buffer, 0.15 M NaCl, saturated CaP soln., pH 7.4, HA	Role of C-terminus in HA adsorption
Le et al., 2006^a^	LRAP and full-length amelogenin (rH174)		Human (recombinant)	ITC, NMR, CD	10 mM HEPES buffer, pH 7.5, pH 4.0 with 10 mM acetate buffer for NMR and CD	Secondary structure, interaction with Ca
Shaw et al., 2008	LRAP		Murine (recombinant)	SS-NMR (REDOR)	NaCl, saturated CaP soln., HA	Secondary structure, dynamics of LRAP interaction with HA
Shaw and Ferris, 2008	LRAP	(+P) and (−P)	Murine (recombinant)	SS-NMR (REDOR)	NaCl, saturated CaP soln., HA	Interaction with HA
Buchko et al., 2010	LRAP and full-length (M180 with His-tag^*^)		Murine (recombinant)	DLS, NMR	pH 3.0, 2% acetic acid, various ionic strength	Quaternary structure, effect of ionic strength
Tarasevich et al., 2010	LRAP	(+P)	Murine (recombinant)	DLS, SWE, AFM	NaCl, saturated CaP soln., or PBS	Quaternary structure, adsorption mechanism on HA
Masica et al., 2011^c^	LRAP	(+P) and (−P)	Murine (recombinant)	SS-NMR (REDOR)	Phosphate buffer, 0.15 M NaCl, saturated CaP soln., pH 7.4, HA	Secondary structure and adsorption mechanism on HA (effect of phosphorylation)
Lu et al., 2013	LRAP	(+P) and (−P)	Murine (recombinant)	SS-NMR (REDOR)	Phosphate buffer, NaCl, saturated CaP soln., HA	Secondary structure and adsorption mechanism on HA (role of phosphorylation, pH, ionic strength)
Tarasevich et al., 2013^c^	LRAP	(+P)	Bovine (recombinant)	SS-NMR (REDOR), SV, NR	Phosphate buffer, NaCl, saturated CaP soln., pH 7.4, HA	Secondary and quaternary structures, adsorption mechanism on HA
Lu et al., 2014	LRAP	(+P)	Murine (recombinant)	SS-NMR (REDOR)	Phosphate buffer, NaCl, saturated CaP soln, pH 7.4, HA and C-HA	Secondary structure and adsorption mechanism on HA or C-HA^*^ (especially in K24-S28 region)
Tarasevich et al., 2015^b^	LRAP	(+P) and (−P)	Murine (recombinant)	SV, SANS, NMR, CD	Phosphate buffer, NaCl, saturated CaP soln	Secondary, tertiary, and quaternary structures

* *CaP, Calcium Phosphate; CD, Circular Dichroism; C-HA, carbonated Hydroxyapatite; DLS, Dynamic Light Scattering; FTIR, Fourier Transform Infrared Spectroscopy; HA, Hydroxyapatite; His-tag, 12 amino acid peptide tag (MRGSHHHHHHGS-); ITC, Isothermal Titration Calorimetry; NMR, Nuclear Magnetic Resonance; NR, Neutron Reflectivity; REDOR, Rotational Echo DOuble Resonance; SAXS, Small Angle X-ray Scattering; SANS, Small Angle Neutron Scattering; SS-NMR, Solid State Nuclear Magnetic resonance; SV, Sedimentation Velocity; SWE, Single wavelength ellipsometry*.

a *This study concluded that non-phosphorylated recombinant human LRAP (58 amino acid residues) had mostly a random coil structure, as we have found in the present study using porcine LRAP. However, the addition of calcium ions did not induce a detectable change in the secondary structure of the human isoform. Although this latter finding may appear to be inconsistent with our present FTIR finding that the addition of calcium induces an increase in random coil structure, it should be noted that even in the absence of calcium we found that the major component (~40%) of the secondary structure of LRAP(−P) is random coil. That is, like Le et al. (2006), our findings similarly indicate that LRAP(−P) predominately exhibits a random coil structure in the absence and presence of calcium*.

b *The findings of this previous study suggest that addition of calcium ion did not affect the amount of α-helix or β-sheet components of LRAP(+P), which appear to be inconsistent with our present study, where a slight decrease in the amount of α-helix component was observed. In this reported study, experiments were carried out in high ionic strength solutions containing 150 mM NaCl, whereas no added background electrolyte was used in the present study. Ionic strength was clearly shown by these authors to affect the zeta potential of LRAP(+P), with a less negative surface charge seen (at neutral pH) at higher ionic strength. Hence, the reduced negative charge on LRAP(+P) may help explain why added calcium did not affect the LRAP(+P) α-helix structure under high ionic strength conditions*.

c *The observed change in LRAP(+P) structure upon HA binding in the present study is consistent with noted previous reports, in which results suggested that amino acid residues K24-K28 of LRAP(+P) molecule had a close to perfect helix structure without HA, but became unfolded to yield a more random structure when adsorbed onto HA*.

The focus of the present study was to investigate the influence of Ser-16 phosphorylation on the LRAP secondary structure using FTIR, because of the marked influence amelogenin phosphorylation has on mineralization *in vitro* (e.g., Kwak et al., 2009; Le Norcy et al., 2011a; Wiedemann-Bidlack et al., 2011) and the potential importance of this finding in the enamel formation process (Margolis et al., 2014). The results of comparative FTIR analyses of LRAP(+P) and LRAP(−P) in solution at pH 7.4 indicate that single-site phosphorylation of LRAP induces clear changes in the secondary structure of the LRAP molecule. The most marked difference is that LRAP(−P) has random coil as the main structure element, whereas LRAP(+P) exhibits more rigid α-helix and β-sheet structures. Our findings also indicate that the presence of calcium ions induces more drastic changes in the secondary structure of LRAP(+P), in comparison to that of LRAP(−P). These general findings mirror our previous TEM and SAXS findings that showed that added calcium had a greater influence on the quaternary and tertiary structures of LRAP(+P), respectively, in comparison to LRAP(−P) (Le Norcy et al., 2011a,b). Furthermore, comparing the changes in the secondary structure of LRAP induced by incubation with ACP or HA, LRAP(+P) showed a completely different pattern of the secondary structures induced by its incubation with ACP from that seen with HA, while LRAP(−P) showed relatively small differences in secondary structure changes following incubation with either HA or ACP.

As shown in Table 1, analyses of the amide I peak reveal that the main structural components of LRAP(−P) are random coil (39.7%) and β-sheets/PPII helix (27.8%). These results are similar to those previously obtained for rP172, which possess the same N- and C-terminal domains of LRAP(−P), along with a large (116 amino acid long) hydrophobic central domain (Lakshminarayanan et al., 2007; Beniash et al., 2012). In sharp contrast to these findings, the main components of LRAP(+P) were found to be α-helix (31.4%) and β-sheets/PPII helix (42.3%), with no evidence of a random coil component. On this basis alone, LRAP(+P) appears to adopt a more ordered secondary structure conformation in solution, in comparison with that found for the non-phosphorylated LRAP(−P).

Differences in amide I spectra (Figure 1F) and FTIR peak analyses (Table 1) also indicate that LRAP(−P) and LRAP(+P) are affected differently by the presence of calcium, as a result of Ser-16 phosphorylation. In the presence of calcium, the random coil component of LRAP(−P) increases substantially (by ~90%) to 74.3%, while more structured elements of β-sheets/PPII helix and β-turn/3(10) helix decrease (by 20%) to yield an overall less rigid structure. This shift in the LRAP(−P) secondary structure in the presence of calcium to a less structured conformation indicates that there are interactions between calcium ions and the non-phosphorylated LRAP(−P). Our previous studies using SAXS, DLS, and TEM showed that addition of calcium to solutions of LRAP(−P) did not change LRAP's tendency to aggregate and form nanospheres in terms of their particle size (Le Norcy et al., 2011a), and had little effect on its globular protein structure observed using SAXS (Le Norcy et al., 2011b). Hence, the observed shift from β-sheet to a less rigid random coil conformation by addition of calcium ions does not appear to affect the tertiary structure of LRAP(−P) or its aggregation and tendency to form nanospheres. This result is in reasonable agreement with a previous study (Le et al., 2006) using circular dichroism (CD), in which it is concluded that non-phosphorylated recombinant human LRAP (58 amino acid residues) had mostly a random coil structure (see Table 4, footnote a).

Interestingly, LRAP(+P) in the presence of calcium ions induces more prominent conformational changes in comparison to the results for LRAP(−P) solutions, as is indicated by the relative magnitude of changes in the overall amide I peak shape (see Figure 1F). As shown in Table 1, LRAP(+P) once again yields a less rigid structure in the presence of calcium, indicated by a reduction in α-helix (from 31.4 to 21.2%) that is offset by an increase in β-turn/3(10) helix (from 11.5 to 21.0%), with essentially no change in the level of β-sheets/PPII helix components (42.3–44.4%). Our FTIR peak analyses also showed that LRAP(+P) in the presence of calcium ions, as in the absence of calcium, exhibits a lack of random coil conformation, unlike that seen with LRAP(−P). Although a slight decrease in the amount of α-helix component was observed, this finding may appear to be inconsistent with earlier CD findings suggesting that addition of calcium ion did not affect the amount of α-helix or β-sheet components of LRAP(+P) (Tarasevich et al., 2015) due to the fact that the latter study was carried out under different experimental conditions (see Table 4, footnote b). Once again, our present findings that the secondary structure of LRAP(+P) is affected substantially more by the addition of calcium ions in comparison to LRAP(−P) parallels our previous results using TEM and SAXS, which show that the assembly/aggregation (Le Norcy et al., 2011a) and the folding (Le Norcy et al., 2011b) of LRAP(+P), respectfully, are similarly more affected by the addition of calcium ions in comparison to LRAP(−P).

Our collective findings, which demonstrate that the presence of a single phosphate group at Ser-16 significantly affects the secondary structure of LRAP in solution and upon subsequent interactions with calcium ions, support the basis of our hypothesis that phosphorylation of this highly-conserved amino acid in an equally conserved context for phosphorylation by Golgi casein kinase induces conformational changes that could critically affect amelogenin's capacity to interact with forming calcium phosphate mineral phases. To explore this idea further, we examined the effect of the interaction of LRAP with HA and ACP. When the non-phosphorylated LRAP(−P) was incubated with either HA or ACP, the proportion of β-sheet structures increased from ~28 to 43% and 54%, respectively (Table 2), along with a marked reduction in the random coil components (from ~40 to 0% and 15%, respectively). As a result of these similar conformational shifts to more rigid structures, following incubation with both mineral phases, amide I peaks for LRAP(−P) in the presence of HA and ACP were also found to be similar (Figure 2A), with the same relatively sharp peak maxima at ~1,620 cm^−1^. The shape of the spectra, however, were found to differ slightly at ~1,650 cm^−1^, most likely due to different amounts of the α-helix component (1,650 cm^−1^) of LRAP(−P) observed following incubation with HA (~19%) in comparison to that observed with ACP (0%) (Table 2).

In contrast to that observed with LRAP(−P), the phosphorylated LRAP(+P) incubated with mineral particles showed more substantial differences in amide I peak shapes that further depended on the *nature* of the calcium phosphate phase present, i.e., HA or ACP (Table 2). When LRAP(+P) was incubated with HA, its secondary structure was found to yield a less rigid conformation, as indicated by a *loss* of α-helix components (from 31% in the absence of HA) and a reduction (from 42 to 20%) in β-sheet structure components, along with an appearance of unstructured random coil (from 0 to 23%), and an increase in 3(10) helix/β-turn components (from 0 to 29%). The observed change in LRAP(+P) structure upon HA incubation is consistent with previous reports (Masica et al., 2011; Tarasevich et al., 2013; see Table 4, footnote c). However, in contrast to that seen in the presence of HA, when LRAP(+P) was incubated with ACP, the overall structure became more rigid with a much greater level of β-sheet (from 42 to 61%), while α-helix components remained at a relatively high level (26%), along with an absence of random coil, similar to that found in the absence of added mineral.

It is interesting that LRAP(+P) showed a quite different pattern of interaction with ACP from that seen with HA, while LRAP(−P) showed relatively small differences in secondary structure changes induced by incubation with either HA or ACP. These findings are again consistent with our previous results (Le Norcy et al., 2011a), in which LRAP(−P) and LRAP(+P) were found to exhibit a marked difference in their ability to stabilize forming ACP under conditions that support spontaneous calcium phosphate precipitation. In this previous report, LRAP(−P) did not stabilize ACP but rather guided the formation of aligned bundles of HA crystals, suggesting a weaker interaction between LRAP(−P) and ACP, whereas LRAP(+P) was found to stabilize ACP and prevent its transformation to HA, suggesting a much stronger interaction between LRAP(+P) and ACP. Hence, the observed difference in the reactivity toward ACP between LRAP(−P) and LRAP(+P) appears to be reflected in observed differences in the secondary structure of LRAP caused by the single phosphorylation site.

Based upon our findings on the effect of phosphorylation on the secondary structure of LRAP(−P) and LRAP(+P) in the absence and presence of calcium in solution and upon binding with selected mineral phases, we conclude, as hypothesized, that Ser-16 phosphorylation induces unique secondary structural changes that may enhance the functional capacity of native phosphorylated amelogenin to effectively stabilize the enamel mineral precursor phase, ACP. The biological relevance of our findings is reflected in a recent study (Beniash et al., 2009) that convincingly demonstrates that the initial forming enamel mineral phase in the early secretory stage of amelogenesis to be ACP that subsequently transforms to HA-like enamel mineral crystals. Our present findings provide insight into how phosphorylation can affect the capacity of native (phosphorylated) amelogenins to stabilize this biologically important ACP enamel mineral precursor phase.

## Author contributions

HY contributed data acquisition, analysis, interpretation, and drafting of the manuscript. HCM contributed to conception and design, data analysis and interpretation, and the drafting of the manuscript. EB contributed to consultation of the methodology and data analysis, interpretation, and critically revising the manuscript. YY and JS contributed to purifying, and providing LRAP, and also critically revising the manuscript. All authors gave final approval and agree to be accountable for all aspects of the work.

### Conflict of interest statement

The authors declare that the research was conducted in the absence of any commercial or financial relationships that could be construed as a potential conflict of interest.
